# Sleep duration and plasma leptin concentrations in early pregnancy among lean and overweight/obese women: a cross sectional study

**DOI:** 10.1186/1756-0500-7-20

**Published:** 2014-01-09

**Authors:** Chunfang Qiu, Ihunnaya O Frederick, Tanya K Sorensen, Daniel A Enquobahrie, Michelle A Williams

**Affiliations:** 1 Center for Perinatal Studies, Swedish Medical Center, 1124 Columbia Street, Suite 750, Seattle, WA 98104, USA; 2 Department of Epidemiology, School of Public Health, University of Washington, Seattle, WA, USA; 3 Department of Epidemiology, Harvard School of Public Health, Boston, MA, USA

**Keywords:** Leptin, Overweight, Obesity, Pregnancy, Sleep duration

## Abstract

**Background:**

Early-pregnancy short sleep duration is predictive of gestational diabetes and preeclampsia; mechanisms for these associations are unknown. Leptin, an adipocyte-derived peptide involved in regulating food intake and energy expenditure, may play a role in these observed associations. Given inconsistent reports linking short sleep duration with leptin, and absence of studies among pregnant women, we examined the association of maternal sleep duration with plasma leptin in early pregnancy.

**Methods:**

This cross-sectional study included 830 pregnant women. Plasma leptin was measured in samples collected around 13 weeks gestation. Sleep duration was categorized as: ≤5, 6, 7–8 (reference), and ≥9 hours. Differences in leptin concentrations across categories were estimated using linear regression. Analyses were completed for lean and overweight/obese women.

**Results:**

Overall, women with long sleep duration had elevated plasma leptin (p-value = 0.04). However, leptin concentrations were not statistically significantly elevated in women with a short sleep duration. There was no association of leptin with sleep duration among lean women. Among overweight/obese women, a U-shaped relation between leptin and sleep duration was observed: Mean leptin was elevated (β = 21.96 ng/ml, P < 0.001) among women reporting ≤5 hour of sleep compared with reference group; and women reporting ≥9 hours of sleep also had elevated leptin (β = 4.29 ng/ml, P = 0.09).

**Conclusions:**

Short sleep duration, and to a lesser extent long sleep duration, were associated with elevated leptin among overweight/obese women. These data add some evidence to help understand mechanistic relationships of sleep duration with pregnancy complications.

## Background

The worldwide prevalence of obesity has doubled since 1980 [1-3]; and this epidemic has been paralleled by a trend in reduced sleep duration [4,5]. Mounting evidence from both experimental and observational studies point to short sleep duration as a risk factor for obesity and obesity-related cardiometabolic disorders including metabolic syndrome, type 2 diabetes, and cardiovascular disorders [6,7]. Furthermore results from controlled metabolic studies show that sleep is an important modulator of neuroendocrine function, glucose metabolism and the secretion of appetite regulatory hormones.

Leptin, an adipocyte-derived hormone known to act on hypothalamic receptors to regulate fat mass, reduce food intake, increase energy expenditure, and stimulate thermogenesis [8,9], has recently been proposed as one possible mechanistic link between decreased habitual sleep duration and increased risk of obesity. Two research groups have reported that shorter habitual sleep durations are associated with reductions in fasting plasma leptin concentrations and higher body mass index [10,11]. Additionally, a number of small clinical experimental studies reported decreases in plasma leptin following acute bouts of sleep restriction [12-19]. However, at least two studies have reported no change in leptin [20,21]; and one study has reported increased leptin concentrations [22] following experimental sleep restriction. Hyperleptinaemia is also an important clinical risk factor for adverse pregnancy outcomes such as preeclampsia [23-25] and gestational diabetes mellitus [26]. At present, little is known about modifiable factors that influence leptin concentrations in maternal circulation during pregnancy.

Collectively, inference from these experimental studies, however, have been limited in part by their small sample size (with many having fewer than 12 participants), limited generalizability (with many restricted to healthy young males), and failure to account for changes in energy expenditure and caloric intake during the experimental periods of observations. Additionally, none of the earlier studies sought to evaluate the influence of habitual short sleep duration on leptin concentration in pregnant women. Given this gap in the literature, and given emerging evidence of increased risks of adverse pregnancy outcomes with habitual short sleep duration [27-29] and hyperleptinemia [23-26], we sought to evaluate the extent to which, if at all, maternal self-reports of habitual sleep duration during early pregnancy is associated with early pregnancy plasma leptin concentrations. Given emerging evidence suggesting that women and overweight/obese individuals may be particularly vulnerable to the physiological consequences of sleep restriction [22], we also examined the extent to which observed association differ by maternal pre-pregnancy lean or overweight/obesity status.

## Results

Table 1 shows demographic characteristics of the 830 women in the study sample according to categories of reported habitual sleep duration during early pregnancy. Overall, maternal plasma leptin concentrations were correlated with systolic (*r =* 0.23), diastolic (*r =* 0.18), and mean arterial blood pressure (*r =* 0.22) (all p-values < 0.001- data not shown). Similarly, maternal plasma leptin concentrations were positively and statistically significantly associated with higher plasma glucose concentrations after a 50-gram oral glucose challenge screening test. Collectively, these correlations are consistent with earlier reports of associations of leptin with increased risks of preeclampsia [23-25] and gestational diabetes [26].

**Table 1:** Sociodemographic characteristics of the study population (N = 830) according to categories of self-reported nightly sleep duration during early pregnancy

	**Categories of hours of sleep during pregnancy**			
	**≤ 5 hours**	**6 hour**	**7-8 hours**	**≥9 hours**
**Characteristics**	**N = 28**	**N = 44**	**N = 485**	**N = 273**
Maternal age (years)	29.6 ± 5.6	31.4 ± 4.1	32.0 ± 4.5	31.6 ± 4.6
18-25	6 (21.4)	3 (6.8)	23 (4.7)	9 (3.3)
25-34	16 (57.1)	33 (75.0)	331 (68.3)	193 (70.7)
≥ 35	6 (21.4)	8 (18.2)	131 (27.0)	71 (26.0)
Maternal race/Ethnicity				
Non-Hispanic White	26 (92.9)	37 (84.1)	417 (86.0)	236 (86.5)
African American	0 (0.0)	3 (6.8)	10 (2.0)	3 (1.1)
Other	2 (7.1)	4 (9.1)	58 (12.0)	34 (12.4)
Payment status				
Insurance	21 (75.0)	37 (84.1)	432 (89.1)	245 (89.7)
Medicaid	4 (14.3)	3 (6.8)	9 (1.8)	10 (3.7)
Missing	3 (10.7)	4 (9.1)	44 (9.1)	18 (6.6)
Nulliparous	23 (82.1)	34 (77.3)	408 (84.1)	252 (92.3)
Not married	11 (39.3)	6 (13.6)	54 (11.1)	27 (9.9)
< 12 years of education	3 (10.7)	1 (2.3)	27 (5.6)	10 (3.7)
Smoked during pregnancy	3 (10.7)	2 (4.6)	30 (6.2)	19 (7.0)
Physically inactive during pregnancy	8 (28.6)	5 (11.4)	81 (16.7)	46 (16.9)
Pre-pregnancy BMI	23.0 ± 4.8	22.3 ± 4.0	23.2 ± 4.8	23.7 ± 5.3
(kg/m^2^)				
< 18.5	2 (7.1)	4 (9.1)	20 (4.1)	9 (3.3)
18.5 – 24.9	21 (75.0)	30 (68.2)	359 (74.0)	196 (71.8)
25-29.9	2 (7.1)	8 (18.2)	68 (14.0)	44 (16.1)
≥ 30.0	3 (10.7)	2 (4.5)	38 (7.9)	24 (8.8)
Mean systolic blood pressure in first trimester (mmHg)	114.2 ± 10.4	114.3 ± 9.4	112.5 ± 10.0	112.6 ± 10.2
Mean diastolic blood pressure in first trimester (mmHg)	69.2 ± 6.3	69.2 ± 7.0	70.3 ± 7.2	69.8 ± 7.0
Mean arterial blood pressure in first trimester (mmHg)	84.2 ± 6.9	84.2 ± 7.0	84.4 ± 7.3	84.1 ± 7.2
Gestational age at blood collection (weeks)	12.9 ± 3.0	14.5 ± 3.3	13.7 ± 3.5	13.6 ± 3.2

Data presented as mean ± SD or number (%).

Mean leptin concentrations were slightly higher among groups of women classified as having short (≤5 hours) and long (≥9 hours) sleep duration during early pregnancy (Table 2). Mean leptin concentrations were lowest for women who reported sleeping 7–8 hours per night during early pregnancy (i.e., the reference group). Compared with the reference group, women who reported sleeping ≤5 hours per night had a statistically non-significant elevation in plasma leptin (27.3 vs. 24.3 ng/ml, p-value = 0.43). Women who reported sleeping ≥9 hours per night during early pregnancy, as compared with the reference group had elevated leptin concentrations (27.0 vs. 24.3 ng/ml, p-value = 0.04).

**Table 2:** Distribution of plasma leptin concentrations according to maternal self-reported nightly sleep duration during early pregnancy

**Early pregnancy**	**Hours of sleep during pregnancy**			
**Plasma leptin (ng/ml)**	**≤5**	**6**	**7-8**	**≥9**
**All women (N)**	*28*	*44*	*485*	*273*
Mean ± SD	27.3 ±19.7	24.3 ± 16.8	24.3 ± 17.1	27.0 ± 17.3
	(p* = 0.43)	(p* = 0.98)	(Reference)	(p* = 0.04)
^**^Lean women (N)	*23*	*34*	*379*	*205*
Mean ± SD	20.6 ± 14.1	19.9 ± 13.6	20.5 ± 14.7	21.7 ± 13.4
	(p* = 0.98)	(p* = 0.82)	(Reference)	(p* = 0.31)
^**^Overweight/obese women (N)	*5*	*10*	*106*	*68*
Mean ± SD	58.3 ± 8.0	39.1 ± 18.8	38.2 ± 18.6	43.0 ± 18.2
	(p* < 0.001)	(p* = 0.98)	(Reference)	(p* = 0.09)

^*^Pair-wise T-test p-value (each sleep duration group compared to reference group of 7–8 hours).

^**^Lean women (BMI <25 kg/m^2^); Overweight/Obese women (BMI ≥25 kg/m^2^).

As shown in the bottom two panels in Table 2, the association between maternal sleep duration and leptin concentrations differed according to maternal pre-pregnancy lean and overweight/obesity status. After stratifying the study population according to lean (BMI <25 kg/m^2^) and overweight/obese women (≥25 kg/m^2^), we noted no association of sleep duration with leptin concentrations among lean women. However, among overweight women, we noted a pronounced U-shaped relationship between plasma leptin and sleep duration. Overweight/obese women who reported sleeping ≤5 hours per night during early pregnancy had substantially higher plasma leptin concentrations as compared with those who reported sleeping 7–8 hours per night (58.3 vs. 38.2 ng/ml, p-value <0.001). Compared with women who reported sleeping 7–8 hours per night (i.e., the reference group), those women who reported sleeping ≥9 hours per night had increased leptin concentrations, though the increase did not reach statistical significance (43.0 vs. 38.2 ng/ml, p-value = 0.09). Given evidence of different associations of sleep duration with leptin concentration, all further analyses were completed for lean (BMI <25 kg/m^2^) and overweight/obese (≥25 kg/m^2^) women separately.

As shown in Table 3, associations between leptin concentration and short sleep duration among overweight/obese women remained after adjustment for potential confounding by maternal age, race/ethnicity, and marital status (Model 1). Notably, the associations remained statistically significant, though somewhat attenuated, after maternal pre-pregnancy BMI was included in the multivariable model (Model 2). After controlling for all confounders, short sleep duration during pregnancy (≤5 hours per night) was associated with higher plasma concentrations on average (mean ± stand error [SE] 21.96 ± 5.30 ng/ml, p <0.001). The fully adjusted model explained 25% of the variance in leptin concentration (adjusted R^2^ = 0.25). Overweight/obese women who reported sleeping ≥9 hours per night also had elevated mean leptin concentrations (mean ± SE 4.29 ± 2.62 ng/ml, p = 0.09) though this association did not reach statistical significance. Consistent with univariate analyses, we observed no evidence of an association between sleep duration and leptin concentrations among lean women (BMI <25 kg/m^2^). Further adjusting for blood pressure in the 1st trimester did not change the estimates.

**Table 3:** Relationship between maternal self-reported hours of nightly sleep duration and plasma leptin concentrations in early pregnancy: estimates linear regression coefficients for the participants of the Omega Study, Seattle and Tacoma, Washington, 1996-2000

**Hours of sleep during pregnancy**	**N**	**MODEL 1**				**MODEL 2**			
		**β**	**SE (β)**	**95% CI**	**P-value**	**β**	**SE (β)**	**95% CI**	**P-value**
**All women**									
≤5	28	4.38	3.76	(−2.99, 11.76)	0.24	4.77	2.88	(−0.89, 10.43)	0.10
6	44	−0.05	2.56	(−5.08, 4.98)	0.99	1.85	2.07	(−2.21, 5.91)	0.37
7-8	485	Reference				Reference			
≥9	273	2.60	1.30	(0.04, 5.16)	0.05	1.77	1.05	(−0.30, 3.83)	0.09
Adjusted R^2^		0.02				0.34			
**Lean women**									
≤5	23	1.24	2.85	(−4.35,6.83)	0.66	1.81	2.84	(−3.76, 7.38)	0.52
6	34	−0.58	2.36	(−5.23, 4.06)	0.81	1.47	2.26	(−2.96, 5.91)	0.51
7-8	379	Reference				Reference			
≥9	205	1.10	1.18	(−1.22, 3.42)	0.35	0.86	1.09	(−1.28, 3.00)	0.43
Adjusted R^2^		0.02				0.15			
**Overweight/obese women**									
≤5	5	24.99	4.06	(16.97, 33.01)	<0.001	21.96	5.30	(11.50, 32.43)	<0.001
6	10	1.40	5.47	(−9.39, 12.20)	0.80	3.95	4.86	(−5.63, 13.53)	0.42
7-8	106	Reference				Reference			
≥9	68	4.61	2.88	(−1.07, 10.30)	0.11	4.29	2.62	(−0.87, 9.46)	0.09
Adjusted R^2^		0.07				0.25			

Separate models were fit for all, lean (BMI <25 kg/m^2^), and overweight BMI ≥25 kg/m^2^) women, respectively.

Model 1: reported coefficients adjusted for maternal age, race/ethnicity, and marital status; and Model 2: Model 1 + maternal pre-pregnancy body mass index.

*β*, Estimated coefficient; *SE (β)*, Standard error of the estimated coefficient; *95% CI*, 95% confidence interval.

**Figure 1:**
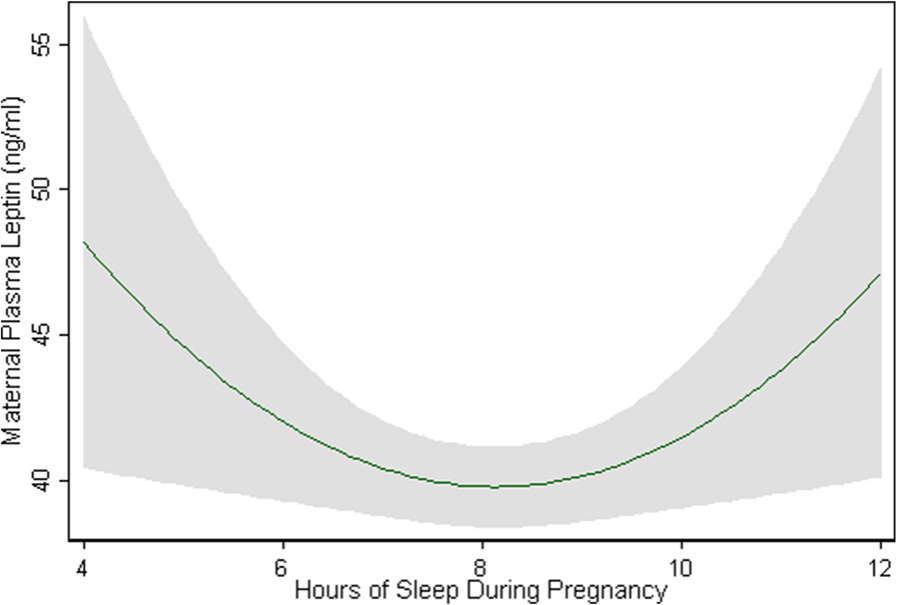
Two-way quadratic prediction plots with 95% confidence interval (gray shade) of predicted maternal plasma leptin concentrations (green line) according to hours of sleep during early pregnancy after adjusting for maternal age, race, marital status and pre-pregnancy BMI.

We next sought to further explore the curvilinear association between leptin concentrations and sleep duration according to sleep duration among overweight/obese women. There was a U-shaped relationship between hours of sleep and leptin concentrations during early pregnancy (Figure 1). However, the quadratic term for sleep duration was not statistically significant (p = 0.18) in the adjusted model that includes maternal age, race, marital status and pre-pregnancy BMI.

## Discussion

In this cross sectional study, we observed a U-shaped relationship between sleep duration and leptin concentrations during early pregnancy among overweight/obese women. In this population, women who reported sleeping ≤5 hours per night had the highest plasma leptin concentrations in early pregnancy (even after additional adjustment for pre-pregnancy BMI). No such relationship was observed among lean women.

We are unaware of published reports concerning the association of sleep duration and leptin concentrations in pregnant women. Our findings, however, are in general agreement with results from some [22,30,31], though not all [10,18,32], studies that have assessed leptin concentrations in relation to habitual sleep duration or acute sleep deprivation among men and non-pregnant women.

In an experimental study of 21 men and women (instructed to not change their usual diet and allowed to choose their meals from a standard meal), Pejovic and colleagues [30] found a 14% increase in 24-hour leptin levels after one night of sleep deprivation. In a recent experimental study involving 136 participants exposed to 5 consecutive nights of restricted sleep (4 hours of time in bed), Simpson and colleagues noted that plasma leptin concentrations were statistically significantly increased during the sleep deprived state as compared with baseline values [22]. In stratified analyses the authors noted that increases in leptin concentrations were statistically significantly higher among women and participants with higher BMI as compared with men and those with lower BMI, respectively [22]. Our results are in agreement with these experimental studies, particularly those that assess sleep duration and leptin associations with consideration of possible heterogeneity according to BMI.

Our findings, however, are not in agreement with other earlier studies [10,18,32]. For example, in their short term partial sleep deprivation study, Spiegel and colleagues reported short sleep was associated with reduction in leptin concentrations among lean men on a controlled diet with constant glucose infusion [18]. Additionally, in a cross sectional analysis from data based on the Wisconsin Sleep Cohort, investigators observed reduced leptin concentrations in relation to reduced habitual sleep duration [10]. In a recent study of 80 obese men and pre-menopausal women, Knutson and colleagues observed no statistically significant associations of relative leptin level (leptin concentration divided by body fat percentage) with habitual sleep duration [32].

Several limitations merit discussion and consideration. First, the cross sectional design of our study and the fact that we did not directly measure maternal sleep duration during pregnancy limited our ability to infer the temporal relationship between sleep duration (or sleep quality), adipose tissue mass and plasma leptin concentrations in early pregnancy. Prospective studies of pregnant women that include objective measures of sleep duration and maternal adipose tissue mass are needed to demonstrate more conclusively these potential causal relationships. Second, a single measurement of plasma leptin may be susceptible to short-term variations, and thus is not likely to provide a time-integrated measure of maternal leptin status during early pregnancy. Consequently, results from our study, with only one measurement of plasma leptin, may have been biased. Longitudinal studies with serial measurements of maternal plasma leptin concentrations are needed to expand upon our current findings. Third, measurement error from the use of self-reported sleep duration is likely to have occurred. However, this error is unlikely to have systematically biased our findings, because the reporting error is not associated with the laboratory determination of maternal plasma leptin concentrations. Misclassification of maternal self-reported sleep duration (unrelated to our laboratory measures of maternal plasma leptin concentrations) would have served to underestimate the true association between the two covariates. Fourth, diurnal variation in leptin concentration may have influenced our results. Because subjects were recruited and enrolled while they attended obstetric clinics to receive standard prenatal care, and because prolonged fasting is contraindicated during pregnancy, we were restricted to measuring leptin in non-fasting blood samples. Finally, the limitation of the relative small sample size in the category of overweight or obese women having short sleep duration must be considered when interpreting our study results.

The identification of leptin as a regulated secreted peptide from adipocytes was a key development in the identification of adipose tissue as an endocrine organ [8]. Despite intense research, the precise metabolic and molecular mechanisms by which leptin secretion is regulated remain incompletely understood. Our findings along with those of others [22] suggest that sleep may have a role in regulation of leptin, particularly in overweight or obese individuals. Given the central role leptin plays in regulating energy homeostasis through central and peripheral mechanisms, and that pregnancy represents a period of profound alterations in glucose homeostasis and lipid metabolism, it stands to reason that factors (such as sleep duration) that may influence leptin concentrations in the non-pregnant state may also play a role during pregnancy. Our findings suggest that alterations in maternal leptin concentrations may be related to habitual sleep duration in early pregnancy. Evaluation of alterations in leptin concentrations according to changes in maternal sleep duration, for example, may be one way of objectively assessing the physiological impact of interventions designed to assess behavioral interventions designed to improve maternal sleep habits and sleep hygiene during pregnancy.

Hyperleptinaemia is an important clinical risk factor for preeclampsia [23-25] and gestational diabetes mellitus [26]. Both disorders are associated with maternal adult weight gain and pre-pregnancy weight gain [33-35]. At present, little is known about modifiable factors that influence leptin concentrations in maternal circulation during pregnancy.

## Conclusions

Our findings suggest that sleep duration, a modifiable factor, and influences leptin concentrations in early pregnancy, particularly among overweight and obese women. If confirmed, improved sleep habits, resulting in improvements in sleep duration and quality, before and during pregnancy may well be one important component in lifestyle programs and strategies aimed towards disease prevention and health promotion in all populations, including pregnant women.

## Methods

### Study design and population

This analysis uses data initially gathered for the Omega Study, a prospective study designed to examine the metabolic and dietary predictors of preeclampsia, gestational diabetes, and other pregnancy outcomes. The study population was drawn from women attending prenatal care at clinics affiliated with Swedish Medical Center and Tacoma General Hospital in Seattle and Tacoma, Washington, respectively. Recruiting began in December 1996. Women who initiated prenatal care before 16 weeks gestation were eligible to participate. Women were ineligible if they were younger than 18 years of age, did not speak and read English, did not plan to carry the pregnancy to term or deliver at either of the two research hospitals, and/or were past 16 weeks gestation.

Enrolled subjects were asked to participate in an interview regarding sociodemographic and lifestyle characteristics and medical and reproductive histories. Non-fasting blood and urine specimens were collected, processed, and stored during early pregnancy. Pregnancy outcome information was abstracted from labor, delivery, and medical records after the estimated delivery date. The procedures used in this study were in agreement with the protocols approved by the Institutional Review Boards of Swedish Medical Center and Tacoma General Hospital. All participants provided written informed consent.

### Analytical population

The analytical population is derived from participants who enrolled in the Omega Study between 1996 and 2000. During this period, 1,219 eligible women were approached, and 1,000 (82 percent) agreed to participate. A total of 91 women with pre-gestational chronic hypertension and/or multifetal pregnancies were excluded from the current analysis. Also excluded were 59 women with missing leptin concentration values, and 20 women with incomplete information on sleep duration during pregnancy. Thus, 830 women remained for analysis.

### Data collection and sleep duration assessment

At the time of enrollment in the Omega study (12.7 weeks gestation, on average), a 45 to 60 minute structured questionnaire was administered by a trained interviewer. Information on maternal sociodemographic, medical, reproductive, and lifestyle characteristics including average number of hours of sleep during early pregnancy was collected. Maternal average nightly sleep duration during pregnancy was ascertained by asking women the following question: “Since becoming pregnant, how many hours per night do you sleep?” Responses were reported as integers. Following the epidemiological literature of sleep duration in adults, particularly available literature focused on pregnant women, we classified participants into 4 sleep duration categories: ≤5 hours (short duration), 6 hours (intermediate duration), 7–8 hours (normal duration or reference group), and ≥9 hours (long duration), respectively.

### Blood collection and plasma leptin measurements

At or near the time of interview (13.2 weeks gestation, on average), a non-fasting blood sample was collected. Blood was drawn into lavender-top vacutainer tubes containing K_3_-EDTA (1 mg/ml). The tube was centrifuged at 850 g for 20 minutes at 4°C to separate red cells, white cells, and plasma. Fractions were aliquoted and stored at −80°C until analysis. Plasma leptin concentrations were measured using an enzyme immunoassay (Diagnostic Systems Laboratory, Inc., Webster, Texas, USA) with the intra- and inter-assay coefficients of variation both <8%. All assays were completed without knowledge of maternal characteristics.

### Statistical analyses

We examined the distributions of maternal sociodemographic, reproductive, and medical characteristics according to four sleep duration categories. We then examined distributions of plasma leptin concentrations and found them to be approximately normal. We therefore reported mean leptin concentrations across sleep duration categories. Spearman’s rank correlation coefficients were calculated to quantify associations between maternal early pregnancy plasma leptin concentrations with first trimester systolic, diastolic and mean arterial blood pressure. Correlations were also estimated to quantify the association of leptin concentrations with plasma glucose concentration after a 50-gram oral glucose challenge.

Multivariable linear regression analyses with robust variances [36] were performed to evaluate the association between sleep duration categories and plasma leptin concentrations. To assess confounding, we entered covariates in Table 1 into a linear regression model sequentially, and then compared the unadjusted and adjusted regression coefficients for sleep duration categories [37]. Final models included covariates that altered unadjusted coefficients for by 10% or more, as well as covariates of *a priori* interest (i.e., maternal age). The following covariates were considered as confounders in the final models: maternal age (<25, 25–34, and ≥35 years), race/ethnicity (White, African American, and Other), marital status (married vs. not married) pre-pregnancy body mass index (BMI). Smoking was found not to be the confounder in this analysis. To assess the potential modifying effects of maternal lean (<25 kg/m^2^) and overweight/obesity (≥25 kg/m^2^) status on the relationships between sleep duration and leptin concentrations, we fit separate linear regression models for each group. We also fit linear regression models with interaction terms between sleep duration and maternal pre-pregnancy BMI. Adjusted R^2^ values were calculated to measure the explanatory power of each model, adjusted for degrees of freedom.

Next, to examine the nonlinear relationship of plasma leptin concentrations and sleep duration in overweight pregnant women, we treated the sleep hours as continuous variable and fit a quadratic model by adding a quadratic term (sleep hours squared) plus a linear term in the regression models. All analyses were performed using Stata 9.0 statistical software (Stata, College Station, Texas, USA). All continuous variables are presented as mean ± standard deviation (SD), unless otherwise specified. All reported p-values are two-tailed.

### Details of ethics approval

The local institution as stated in the Methods section has approved human experimentation. Institutional Review Board #2505S-94 (Continuing Review Progress Report) was obtained on 12-20-2011. The written consent has been obtained from all patients participating in current study.

## Competing interest

The authors declare that they have no competing interests.

## Authors’ contributions

CQ and MAW had full access to all the data in the study and take responsibility for the integrity of the data, the accuracy of the data analysis, and the decision to submit for publication. CQ analyzed the data. CQ and MAW drafted the manuscript. IOF, TKS and DAE reviewed and edited the manuscript. MAW conceived, designed and obtained funding for the study. All authors interpreted the data, critically revised the draft for important intellectual content, and gave final approval of the manuscript to be published.
